# The IFN-induced protein IFI27 binds MDA5 and counteracts its activation after SARS-CoV-2 infection

**DOI:** 10.3389/fcimb.2024.1470924

**Published:** 2024-10-04

**Authors:** Vanessa Rivero, Julia Carrión-Cruz, Darío López-García, Marta L. DeDiego

**Affiliations:** Department of Molecular and Cell Biology, Centro Nacional de Biotecnología (CNB-CSIC), Campus Universidad Autónoma de Madrid, Madrid, Spain

**Keywords:** IFI27, MDA5, innate immune responses, interferon, inflammation, SARS-CoV-2, innate immunity, RIG-I-like receptors

## Abstract

Innate immune responses are induced after viral infections, being these responses essential to establish an antiviral response in the host. The RIG-I-like receptors (RLRs), RIG-I and MDA5 are pivotal for virus detection by recognizing viral RNAs in the cytoplasm of infected cells, initiating these responses. However, since excessive responses can have a negative effect on the host, regulatory feedback mechanisms are needed. In this work, we describe that IFN alpha-inducible protein 27 (IFI27) co-immunoprecipitates with melanoma differentiation-associated protein 5 (MDA5), being this interaction likely mediated by RNAs. In addition, by using IFI27 overexpression, knock-out, and knock-down cells, we show that IFI27 inhibits MDA5 oligomerization and activation, counteracting the innate immune responses induced after SARS-CoV-2 infections or after polyinosinic-polycytidylic acid (poly(I:C)) transfection. Furthermore, our data indicate that IFI27 competes with MDA5 for poly(I:C) binding, providing a likely explanation for the effect of IFI27 in inhibiting MDA5 activation. This new function of IFI27 could be used to design target-driven compounds to treat diseases associated with an exacerbated induction of innate immune responses, such as those induced by SARS-CoV-2.

## Introduction

The host innate immune system detects viral infections, relying on pattern recognition receptors (PRRs), which sense pathogen-associated molecular patterns (PAMPs), such as viral RNAs. The retinoic acid-inducible gene I (RIG-I)-like receptors (RLRs), RIG-I and melanoma differentiation-associated protein 5 (MDA5) are pivotal for virus detection by recognizing viral RNAs in the cytoplasm of infected cells. Severe Acute Respiratory Syndrome coronavirus 2 (SARS-CoV-2) is a single-stranded, positive-sense, RNA virus belonging to the *Coronaviridae* family. SARS-CoV-2 replication triggers MDA5-dependent innate immune responses (Rebendenne et al., 2021; Sampaio et al., 2021; Thorne et al., 2021; Yin et al., 2021). RIG-I and MDA5 contain a DExD/H-box RNA helicase domain and a C-terminal domain (CTD), both responsible for RNA binding. In addition, RIG-I and MDA5 have two N-terminal caspase activation and recruitment domains (CARDs). Upon activation of RLRs by RNA binding, the proteins undergo conformational changes, and expose their CARDs. The CARDs from multiple MDA5 or RIG-I proteins oligomerize and become accessible for interaction with the adaptor mitochondrial antiviral signaling (MAVS) protein, ultimately leading to the induction of innate immune responses (Dias Junior et al., 2019).

RLR activation induces the production of inflammatory cytokines and type I and III interferons (IFNs), which, in turn, propagate antiviral signaling by upregulating IFN-stimulated genes (ISGs). Hundreds of ISGs have already been described, and they have an enormous variety of functions. Several ISGs have a clear antiviral function, but there are also ISGs that are able to prevent an excessive immune response, since exacerbated innate immune responses can be deleterious to the host, playing a regulatory role (Richards and Macdonald, 2011). As an example, we previously described the molecular mechanisms underlying negative modulation of IFN responses mediated by the ISGs IFI6, IFI27, IFI44 and IFI44L that facilitate virus replication (DeDiego et al., 2019b, 2019a; Villamayor et al., 2023a, 2023b).

IFI27 (also known as ISG12a) encodes a 122-amino acid hydrophobic protein of 12 kDa, which has an N-terminal mitochondrial targeting sequence. It belongs to the FAM14 family, comprising four genes in humans (IFI6 or G1P3, IFI27 or ISG12a, IFI27L2 or ISG12b and IFI27L1 or ISG12c) (Cheriyath et al., 2011). We previously showed that IFI27 negatively modulates IFN responses, positively affecting influenza A virus (IAV) and SARS-CoV-2 replication (Villamayor et al., 2023a). We also showed that IFI27 interacts with nucleic acids and RIG-I, being the interaction of IFI27 with RIG-I most likely mediated through RNA binding, leading to impaired RIG-I activation (Villamayor et al., 2023a). IFI27 is likely a pro-apoptotic factor since this protein associates with or inserts into the mitochondrial membrane, and it contributes to IFN-dependent perturbation of the mitochondrial membrane permeability (Rosebeck and Leaman, 2008). Furthermore, IFI27 encodes two putative Bcl-2 homology (BH) 3-like motifs and sensitizes cells to RNF and BH3 mimetic-induced apoptosis (Gytz et al., 2017); and it augmented TNF-related apoptosis-inducing ligand (TRAIL)-induced apoptosis through intrinsic apoptotic pathway (Liu et al., 2019).

IFI27 has shown an antiviral effect against Hepatitis C Virus (HCV). The molecular mechanism involves the ubiquitin-dependent degradation of HCV NS5A protein, as it is able to promote the association of the S-phase kinase-associated protein 2 (SKP2), which is an ubiquitin E3 ligase, to HCV NS5A, mediating its degradation via the proteasome (Xue et al., 2016). Similarly, IFI27 inhibits hepatitis B virus (HBV) gene expression and replication, through an unknown mechanism (Ullah et al., 2021). High IFI27 levels have been detected in blood of infants hospitalized with Respiratory Syncytial Virus (RSV) (Fjaerli et al., 2006), and after influenza infections (Tang et al., 2017; Ravi et al., 2022). After RSV infection, IFI27 expression has been positively associated with more severe cases, more requirements of mechanical ventilation, more frequent hospitalization, and longer hospital stays in preterm infants, proposing the use of IFI27 as a biomarker of RSV disease severity and outcome (Gao et al., 2021). Furthermore, IFI27 expression is upregulated in the blood of SARS-CoV-2 positive patients (Gupta et al., 2021; Huang et al., 2021; Shojaei et al., 2022), and IFI27 is upregulated in the respiratory tract of COVID-19 patients (Mick et al., 2020; Kulasinghe et al., 2022; Shojaei et al., 2022). However, the upregulation of IFI27 in virus-infected subjects could just reflect host innate immune responses elicited towards the viral replication.

In this work we describe, for the first time, that IFI27 interacts with MDA5, being this interaction likely mediated by RNAs. In addition, we show that IFI27 inhibits MDA5 oligomerization and activation, counteracting the innate immune responses induced after SARS-CoV-2 infections or after polyinosinic-polycytidylic acid (poly(I:C)) transfection. This new function of IFI27 could be used to design target-driven compounds to treat diseases associated with an exacerbated induction of innate immune responses and to inhibit viral infections, such as SARS-CoV-2.

## Materials and methods

### Cells and viruses

Human embryonic kidney 293T (ATCC CRL-11268), human lung epithelial carcinoma A549 (ATCC CCL-185), and African green monkey kidney epithelial Vero E6 (ATCC CRL-1586) cells, were kindly provided by Prof. Luis Enjuanes (Centro Nacional de Biotecnología, CNB-CSIC, Spain). All the cells were grown at 37°C in air enriched with 5% CO_2_ using Dulbecco’s modified Eagle’s medium (DMEM, Gibco) supplemented with 10% fetal bovine serum (Gibco), and 50 μg/ml gentamicin (Gibco). 293T cells and A549 cells overexpressing human ACE2 (293T-ACE2 and A549-ACE2) were obtained by transducing the cells with a retrovirus expressing human ACE2 and a blasticidin resistance gene (kindly provided by Pablo Gastaminza, National Center for Biotechnology, Madrid, Spain). The A549-ACE2 cells knocked-out (KO) for IFI27 were generated by our group and previously described (Villamayor et al., 2023a). The 293T-ACE2 and A549-ACE2 cells were grown in the same media containing 5 and 2.5 μg/ml of blasticidin (ThermoFisher Scientific), respectively.

The recombinant Vesicular Stomatitis Virus, Indiana strain, encoding the green fluorescent protein, GFP (rVSV-GFP) (Stojdl et al., 2003), and the SARS-CoV-2 (kindly provided by prof. Luis Enjuanes, at Centro Nacional de Biotecnología, CNB-CSIC, Spain) (Wang et al., 2023) were grown in Vero E6 cells. rVSV-GFP and SARS-CoV-2 were titrated by plaque assay (plaque forming units, PFU/ml) in confluent monolayers of Vero E6 cells seeded in 24-well plates, as previously described (DeDiego et al., 2016; Saiz et al., 2021).

### Plasmids

The polymerase II expression pCAGGS plasmid encoding IFI27 (GenBank accession number NM_001130080.3) C-terminally fused to an HA epitope tag (pCAGGS-IFI27-HA), was generated by our group and previously described (Villamayor et al., 2023a). This plasmid was previously modified to encode a hygromycin resistance gene. The pCAGGS plasmid expressing RIG-I protein (GenBank accession number AF038963.1) fused to a FLAG epitope tag (pCAGGS-RIG-I-FLAG) was previously described (Mibayashi et al., 2007). The pCAGGS plasmid expressing MDA5 protein (GenBank accession number NM_022168.4) fused to a FLAG epitope tag (pCAGGS-MDA5-FLAG) was kindly provided by Luis Martinez-Sobrido (Texas Biomedical Research Institute, San Antonio, Texas, US).

### Generation of A549-ACE2 cells stably expressing IFI27

The pCAGGS-IFI27-HA and pCAGSs-empty plasmids encoding a hygromycin resistance gene were linearized using the restriction enzyme *SalI* (New England biolabs). Then, the linearized plasmids were purified and transfected into IFI27 knock-out A549 cells overexpressing ACE2 (A549-ACE2-IFI27 KO cells using lipofectamine 3000 (ThermoFisher Scientific), for 48 h. At 48 h post-transfection, the transfected cells were selected by growing the cells in the presence of 400 μg/ml hygromycin (ThermoFisher Scientific). The cells were grown in the same hygromycin concentration during the passages.

### Co-immunoprecipitation assays

Human 293T-ACE2 cells (100 mm-plate format) were transiently co-transfected with the plasmids pCAGGS-IFI27-HA, pCAGGS-MDA5-FLAG, pCAGGS-RIG-I and/or the empty pCAGGS plasmid using lipofectamine 3000 (ThermoFisher Scientific), for 24 h. The total amount of transfected DNA plasmid was always the same, as the empty pCAGGS plasmid was co-transfected when needed. Later, cells were left mock-transfected, transfected with poly(I:C) (60 ng/ml) using polyethylenimine (PEI, Polysciences) for an additional 24 h or infected with SARS-CoV-2 (multiplicity of infection, MOI 0.5) during 24 h, and cells were lysed in the coimmunoprecipitation buffer (NaCl 250 mM; EDTA 1 mM; 50 mM TrisHCl, pH 7.5; NP-40 0.5%) containing protease (ThermoFisher Scientific) and phosphatase (Merck) inhibitors, and cleared by centrifugation. Where indicated, cellular lysates were treated with RNaseA (10 U/ml), RNase T1 (400 U/ml) and RNAse III (10 U/ml), for 1 h at 37°C, as previously reported (Laraki et al., 2008; Villamayor et al., 2023a). Cleared cell lysates were incubated overnight at 4°C with the anti-FLAG affinity resin (Sigma-Aldrich, A2220), or the anti-HA affinity resin (Pierce, 26181). Alternatively, A549-ACE2 IFI27 KO cells overexpressing IFI27 (A549-ACE2-IFI27 KO + IFI27-HA) were treated with 60 ng/ml poly(I:C) using PEI for an additional 24 h, and cells were lysed in the coimmunoprecipitation buffer and cleared by centrifugation. Cleared cell lysates were incubated overnight at 4°C with the MDA5 specific antibody (21775-1-AP, Thermo Fisher Scientific) and protein A-sepharose (P3391, Merck) resin (Sigma-Aldrich, A2220). The cellular extracts combined with the affinity resins were washed three times in TBS buffer containing 0.1% SDS. The immunoprecipitated proteins were unbound using 0.1 M glycine buffer at pH 2.4, denatured in loading buffer, and incubated at 95°C, during 5 min. Then, samples were analyzed by electrophoresis and Western blot as described below.

### Western blot assays

Cell lysates and co-immunoprecipitation eluates were mixed with Laemmli sample buffer (Biorad) containing 2.5% β-mercaptoethanol, and heated at 95°C for 5 min, before sodium dodecyl sulfate (SDS)-polyacrylamide gel electrophoresis (PAGE) under denature conditions. For analyzing MDA5 oligomers under semi-denaturating detergent (SDD)-PAGE electrophoresis, cell lysates were mixed with Laemmli sample buffer without β-mercaptoethanol and protein electrophoresis was performed in gels without SDS (4–20% Mini-PROTEAN^®^ TGX™ Precast Protein Gels, Biorad). Proteins were transferred to nitrocellulose membranes (Biorad), and detected using the primary antibodies: anti-SARS-CoV-2 nucleocapsid (N) protein (GeneTex, GTX135357), anti-HA (Sigma-Aldrich, H6908) to detect IFI27-HA tagged protein, anti-FLAG (F3165, Sigma-Aldrich), to detect RIG-I or MDA5-FLAG tagged proteins, anti-MDA5 (21775-1-AP, Thermo Fisher Scientific), anti-GFP (11814460001, Merck), and anti-actin (A4700, Sigma Aldrich). Then, the membranes were incubated with a 1:4,000 dilution of goat anti-rabbit (pAb) or anti-mouse (mAb) IgG antibodies conjugated to horseradish peroxidase (Sigma-Aldrich). Membranes were revealed by chemiluminescence, according to the manufacturer’s recommendations, using the SuperSignal west femto maximum sensitivity substrate (ThermoFisher Scientific).

### Immunofluorescence and confocal microscopy

Confluent monolayers of human 293T-ACE2 cells were grown on sterile glass coverslips (24-well format) and were transiently transfected, using lipofectamine 3000 (ThermoFisher Scientific), with the pCAGGS plasmids expressing MDA5-FLAG and IFI27-HA. At 24 hpt, cells were mock-transfected, transfected with 60 ng/ml poly(I:C) or infected with SARS-CoV-2. Alternatively, confluent monolayers of the A549-ACE2 stably expressing IFI27 (A549-ACE2-IFI27 KO + IFI27-HA) or control cells (A549-ACE2-IFI27 KO + empty) were mock-transfected, transfected with poly(I:C) or infected with SARS-CoV-2. After poly(I:C) transfection or SARS-CoV-2 infection, cells were fixed and permeabilized with 10% formaldehyde and 0.1% Triton-X100 during 20 min at RT. Then, cells were blocked with 10% fetal bovine serum in PBS during 1h at RT. MDA5 was detected with an anti-MDA5 antibody generated in rabbit (21775-1-AP, Thermo Fisher Scientific), and IFI27-HA was detected with anti-HA antibody generated in mouse (sc-7392, Santa Cruz Biotechnology). SARS-CoV-2 infection was detected with an affinity purified anti-N protein antibody generated in rat, using His-tagged SARS-CoV-2 N protein as antigen (González‐Cabaleiro et al., 2024). Coverslips were washed 4 times with PBS and stained with secondary anti-mouse, anti-rabbit and anti-rat Abs conjugated to Alexa Fluor 488, 546 and 647, (Invitrogen), respectively, and nuclei were stained using DAPI (ThermoFisher Scientific), during 45 min at RT. Coverslips were mounted in ProLong Gold antifade reagent (Invitrogen) and analyzed on a Leica STELLARIS 5 confocal microscope. Images were acquired with the same instrument settings and analyzed using the Fiji software.

### Silencing of IFI27

Human A549-ACE2 cells were transfected with a small interfering RNA (siRNA) specific for human IFI27 (ThermoFisher Scientific, s7140), or with the non-targeting (NT) negative control (ThermoFisher Scientific, AM4635), twice, 24 h apart. All siRNAs were transfected at a final concentration of 20 nM, using lipofectamine RNAiMax (ThermoFisher Scientific), according to the manufacturer’s instructions, as previously described (Villamayor et al., 2023a).

### IFN response assays

Human A549-ACE2 cells were transfected with an siRNA specific for IFI27, or the NT control siRNA twice, 24 h apart. Alternatively, parental A549-ACE2 cells and cells specifically knocked-out for IFI27, previously generated (Villamayor et al., 2023a), and the stable IFI27 KO cell lines transfected with the plasmid expressing the IFI27-HA (A549-ACE2-IFI27 KO + IFI27-HA) and the control cells or the empty plasmid (A549-ACE2-IFI27 KO +empty) were seeded. Then, the cells were infected with SARS-CoV-2 (MOI 0.5). Alternatively, the cells were transfected with poly(I:C) (Sigma) using PEI during 24 h. SARS-CoV-2 titers were determined as described above. Alternatively, total RNA was extracted, and RT-qPCRs were performed, as described below. In addition, the cells were seeded and transfected with poly(I:C) using PEI. At 16 h after treatment, cells were infected with rVSV-GFP for 24 h and viral titers in cell culture supernatants were determined in Vero cells as previously described (DeDiego et al., 2019a, 2019b; Villamayor et al., 2023a).

### Quantitative PCR assays

mRNA levels of IFI27, IFNL1, and IFN-induced protein with tetratricopeptide repeats 2 (IFIT2), and CXCL10 in human A549-ACE2 cells were analyzed by qPCR. To this end, total RNAs were extracted using the total RNA extraction kit (Omega Biotek). Retrotranscriptase (RT) reactions were performed using the High Capacity cDNA transcription kit (ThermoFisher Scientific) at 37°C for 2 h, using random primers, and total RNA as template. qPCRs were performed using TaqMan gene expression assays (Applied Biosystems) specific for human IFI27 (Hs01086373_g1), human IFNB1 (Hs02621180_s1), human IFIT2 (Hs00533665_m1), human IFNλ1 (Hs00601677_g1), human CXCL10 (Hs00171042_m1), and human GAPDH (Hs02786624_g1) genes. Quantification was achieved using the threshold cycle (2^−ΔΔ^ *^CT^*) method (Livak and Schmittgen, 2001) and normalized with GAPDH expression levels.

### Binding of MDA5 to poly(I:C) in the presence of IFI27

Human 293T cells (6-well plate format) were transiently transfected with a plasmid expressing MDA5-FLAG alone or in combination with a plasmid expressing IFI27-HA or with a plasmid expressing GFP as a negative control, using lipofectamine 3000 for 24 h. Cells were lysed in coimmunoprecipitation buffer (NaCl 250 mM; EDTA 1 mM; 50 mM Tris-HCl, pH 7.5; NP-40 0.5%) containing protease (ThermoFisher Scientific) and phosphatase (Merck) inhibitors. To prepare poly(I:C)-conjugated agarose beads, 2 mg of poly(C)-conjugated agarose beads (Sigma) per sample were washed five times with Tris-Buffered Saline (TBS) buffer (25 mM Tris, 150 mM NaCl). The beads were then resuspended in buffer containing 50 mM Tris and 50 mM NaCl and incubated overnight with 60 μg of inosinic acid (Sigma). The beads were washed twice with TBS, resuspended in TBS buffer containing 1 mM EDTA and 0.5% Triton X-100, and incubated at 4°C for 2 h with the cellular extracts expressing MDA-5, MDA5 plus IFI27 or GFP. The mixture was washed 4 times with TBS buffer containing 1 mM EDTA and 0.1% Tween 20, and the bound proteins were eluted in loading buffer at 95°C during 5 min. The eluted proteins were analyzed by Western blotting using Abs, as described above.

## Results

### IFI27 interacts with MDA5

**Figure 1:**
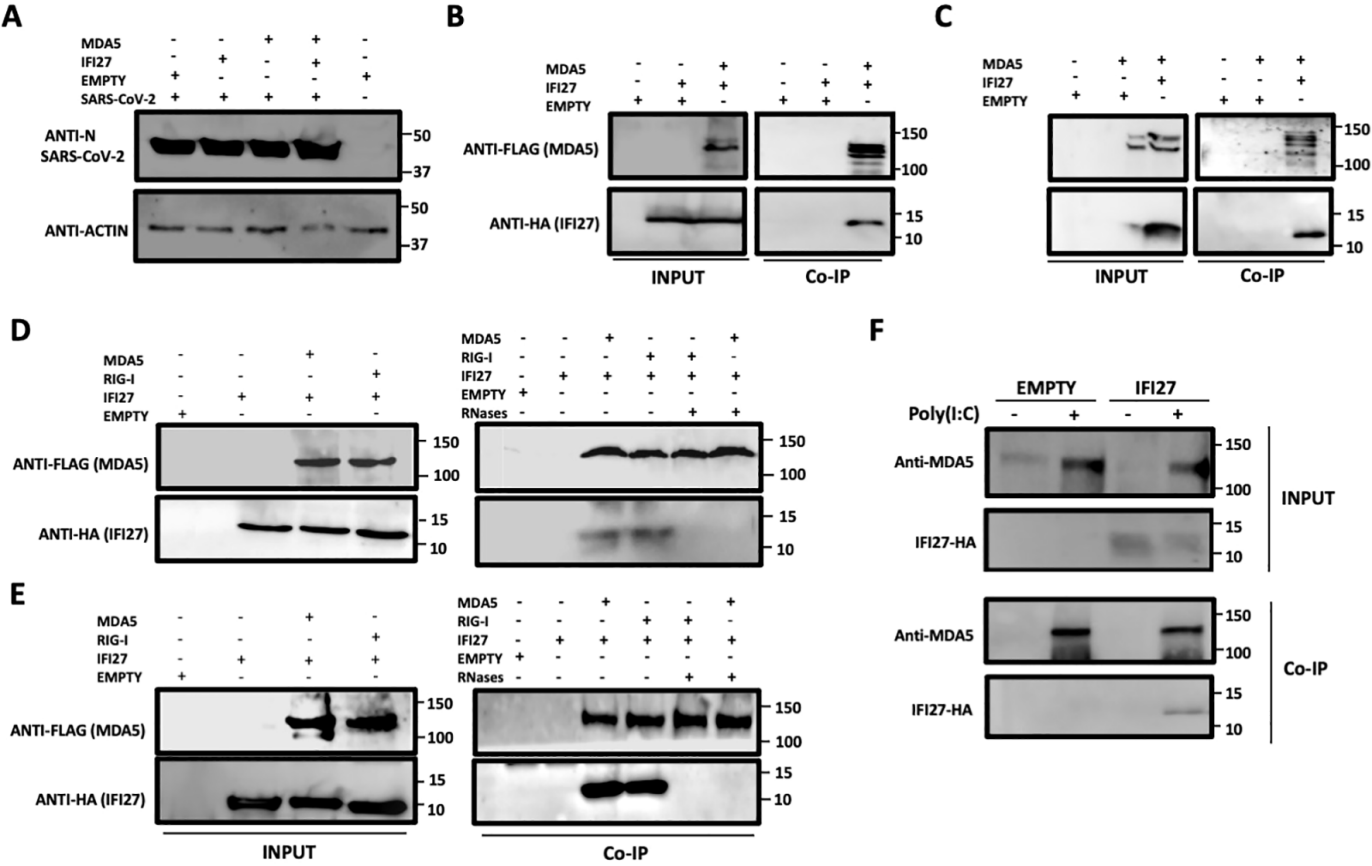
IFI27 binds MDA5. **(A–C)** Human 293T-ACE2 were co-transfected with the pCAGGS plasmids encoding IFI27-HA and MDA5-FLAG, or with empty plasmids. At 24hpt the cells were infected with SARS-CoV-2 for 24h. **(A)** SARS-CoV-2 infection was confirmed by detecting the viral nucleocapsid (N) protein in the cellular extracts by Western blotting using an antibody specific for SARS-CoV-2 N protein (top blots). As loading control, an anti-actin antibody was used (bottom blot). In addition, a cellular extract from mock-infected cells was used. **(B, C)** Co-immunoprecipitation (co-IP) experiments were performed using either an anti-FLAG **(B)** and anti-HA **(C)** antibodies conjugated to agarose beads, to pull down MDA5 or IFI27, respectively. Both proteins were detected by Western blotting using antibodies specific for the HA-tag (to detect IFI27) and anti-MDA5 in the cellular lysates (INPUT) and after the co-IP **(B, C)**. Molecular weight markers are indicated (in kilodaltons) on the right. **(D, E)** 293T-ACE2 cells were co-transfected with the pCAGGS plasmids encoding IFI27-HA, MDA5-FLAG, RIG-I-FLAG or with empty plasmids and then, the cells were left mock-transfected **(D)** or transfected with 60 ng/ml poly(I:C) for 24h **(E)**. After checking that MDA5, RIG-I and IFI27 were expressed correctly (INPUT, left blots in **D**, **E**), cellular extracts were treated with RNases or left untreated and co-immunoprecipitation (Co-IP) experiments using an anti-FLAG antibody conjugated to agarose beads, to pull down MDA5 and RIG-I proteins, were performed. IFI27, MDA5, and RIG-I were detected by Western blotting using antibodies specific for the HA tag (to detect IFI27) or the FLAG tag (to detect RIG-I and MDA5 protein) in the cellular lysates (INPUT) and after the Co-IP. Molecular weight markers are indicated (in kilodaltons) on the right. **(F)** A549-IFI27-KO cells stably overexpressing IFI27 by means of a plasmid were transfected with poly (I:C). At 24 hpt, an immunoprecipitation assay using an anti-MDA5 antibody coupled to protein A-sepharose beads was performed. MDA5 and IFI27 were detected by Western blotting antibodies specific for the HA-tag (to detect IFI27) and anti-MDA5 after the IP. Molecular weight markers are indicated (in kilodaltons) on the right.

We previously showed that IFI27 binds the dsRNA analog poly(I:C) and that IFI27 interacts with the PRR RIG-I, being this last interaction likely mediated by the binding of IFI27 to dsRNAs, such as viral dsRNAs and poly(I:C) (Villamayor et al., 2023a). Given that MDA5 also recognizes viral ssRNAs and dsRNAs, such as SARS-CoV-2 RNAs (Rebendenne et al., 2021; Sampaio et al., 2021; Yin et al., 2021), and poly(I:C) (Gitlin et al., 2006; Kato et al., 2008; Dias Junior et al., 2019), we hypothesized that IFI27 could be directly or indirectly interacting with MDA5, in addition to RIG-I. To test this hypothesis, we transfected 293T cells overexpressing human ACE2 by means of a retrovirus (293T-ACE2) with pCAGGS plasmids expressing IFI27 fused to the HA tag (IFI27-HA) and MDA5 fused to a FLAG tag (MDA5-FLAG). Then, we infected the cells with SARS-CoV-2 and cell extracts were collected. First, we confirmed SARS-CoV-2 infection using the cellular extracts by performing a Western blot with an anti-nucleocapsid (N) protein (**Figure 1A**). Then, the cell extracts were used in an immunoprecipitation assay using agarose beads conjugated to a FLAG antibody (**Figure 1B**) or an anti-HA antibody (**Figure 1C**). Interestingly, MDA5 and IFI27 co-immunoprecipitated together (**Figures 1B, C**) using both antibodies, suggesting a direct or indirect interaction of IFI27 and MDA5 in SARS-CoV-2-infected cells. As controls, after the coimmunoprecipitation using the anti-FLAG antibody, IFI27-HA was not detected when MDA5 was not overexpressed (**Figure 1B**), and using the anti-HA antibody, MDA5-FLAG was not detected when IFI27-HA was not overexpressed (**Figure 1C**).

As MDA5 (Gitlin et al., 2006; Kato et al., 2008; Dias Junior et al., 2019), and IFI27 (Villamayor et al., 2023a) both bind poly(I:C), and we had previously shown that the interaction of RIG-I and IFI27 is mediated by viral RNAs or poly(I:C) (Villamayor et al., 2023a), we analyzed whether IFI27 and MDA5 interact in poly(I:C)-transfected cells and whether this interaction is mediated by poly(I:C). In addition, we analyzed whether these two proteins could interact in mock-infected cells. To this end, 293T-ACE2 cells were transfected with pCAGGS plasmids expressing IFI27-HA and RIG-I-FLAG or with plasmids expressing IFI27-HA and MDA5-FLAG and co-immunoprecipitations were performed using the cells extracts without treatment or after being treated with RNAseT1 and RNaseA (to digest ssRNAs), and with RNAseIII (to digest dsRNAs) (**Figures 1D, E**). In mock-transfected (**Figure 1D**) and poly(I:C)-transfected (**Figure 1E**) cells, the amount of IFI27 protein co-immunoprecipitated with RIG-I (as control) and MDA5 was clearly decreased, almost to undetectable levels, in the cell extracts previously treated with the cocktail of RNAses, compared with the extracts non-treated with RNAses, in which both RIG-I and MDA5 proteins were detected (**Figures 1D, E**). These results confirm that the interaction of IFI27 with RIG-I is RNA-mediated, as we previously described (Villamayor et al., 2023a), and indicate that IFI27 and MDA5 co-immunoprecipitate together as both proteins bind RNA. The results in mock-transfected cells (**Figure 1D**), could imply that after plasmid transfection and MDA5 overexpression, MDA5 could bind cellular RNAs, as previously indicated (Dias Junior et al., 2019; Straub and Sampaio, 2023). However, MDA5 expression levels in non-infected cells or cells non-treated with poly(I:C) is very low, so this pathway is not activated to a high level under basal conditions.

To further analyze the interaction of IFI27 with MDA5, cells overexpressing IFI27-HA were generated. To this end, IFI27 knock-out A549 cells overexpressing ACE2 by means of a retrovirus (A549-ACE2-IFI27 KO cells), previously generated (Villamayor et al., 2023a), were transfected with the pCAGGS plasmid expressing IFI27 fused to an HA tag or with the empty plasmid as control, both of them linearized. Then, the transfected cells were selected in the presence of the hygromycin antibiotic, given that this plasmid had been modified to express a hygromycin-resistance gene. First, the expression of IFI27-HA in the cells generated, was confirmed by Western blot using an antibody specific for the HA tag (**Figure 1F**) and by immunofluorescence (**Supplementary Figure S1**). Later on, the cells were left mock-transfected or the cells were transfected with poly(I:C), cell extracts were collected and a co-immunoprecipitation using an anti-MDA5 antibody and protein A coupled to sepharose beads was performed (**Figure 1F**). The expression of MDA5 was clearly induced in the poly(I:C)-transfected cells, compared to the control cells, as previously described (Yang et al., 2013; Liu et al., 2021). Interestingly, MDA5 and IFI27-HA co-immunoprecipitated together (**Figure 1F**) in the cells transfected with poly(I:C), and not in the mock-transfected cells, likely due to the fact that MDA5 is expressed to very low levels in the non-transfected cells (**Figure 1F**). As control, IFI27 was not detected after the co-immunoprecipitation in the cells non-transfected with poly(I:C). These results further indicate that IFI27 and endogenous MDA5 bind to each other.

**Figure 2:**
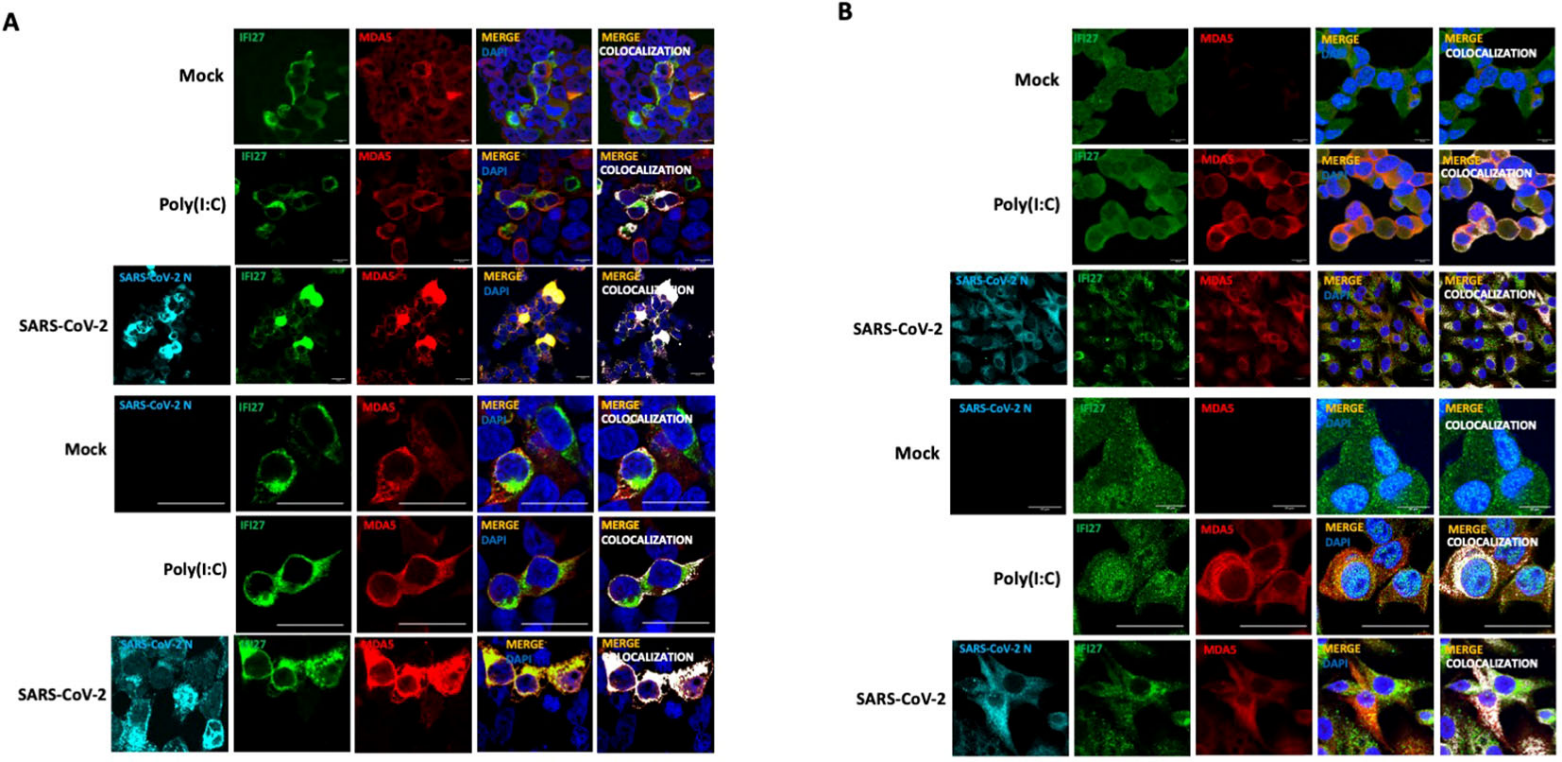
IFI27 and MDA5 colocalize intracellularly. **(A)** The 293T-ACE2 cells were transiently transfected with the pCAGGS plasmids encoding IFI27-HA and MDA5-FLAG and left mock-transfected, transfected with 60 ng/ml poly(I:C) or infected with SARS-CoV-2. at 24 hpt or hpi (for SARS-CoV-2 infection), cells were fixed with paraformaldehyde, and MDA5-FLAG and IFI27-HA proteins were labeled with anti-MDA5 and anti-HA (to detect IFI27) specific antibodies (in red and green, respectively), SARS-CoV-2 N protein was labeled with an anti-N antibody (in cyan), and nuclei were stained with DAPI (in blue). Areas of co-localization of MDA5-FLAG and IFI27-HA proteins appear in yellow-orange and white. Scale bar, 10 μm. **(B)** A549 cells stably expressing IFI27-HA were mock-transfected, transfected with 60 ng/ml poly(I:C) or infected with SARS-CoV-2. At 24 hpt, cells were fixed with paraformaldehyde and MDA5 and IFI27-HA were labeled with anti-MDA5 and anti-HA (to detect IFI27) specific antibodies (in red and green, respectively), SARS-CoV-2 N protein was labeled with an anti-N antibody (in cyan), and nuclei were stained with DAPI (in blue). Areas of co-localization of both proteins appear in orange and white. Scale bar, 10 μm. Two different set of pictures at different magnifications are shown for each condition and cell line.

To study whether IFI27 and MDA5 colocalize intracellularly, supporting their interaction inside the cells, 293T-ACE2 cells were transiently transfected with the pCAGGS plasmids expressing MDA5-FLAG and IFI27-HA, and then left mock-transfected, transfected with poly(I:C) or infected with SARS-CoV-2 during 24 h. SARS-CoV-2 infection was confirmed by using an anti-SARS-CoV-2 N protein antibody (**Figure 2A**). As control, MDA5-FLAG and IFI27-HA expression was not detected in cells non-transfected with the plasmids expressing these fusion proteins (data not shown). A partial co-localization of IFI27 and MDA5 in the cytoplasm, more prominent in the cells transfected with poly(I:C) and SARS-CoV-2, than in the mock-transfected cells was observed (**Figure 2A**), showing a Pearson correlation coefficient (Dunn et al., 2011) for IFI27 and MDA5 fluorescent signals of 0.43 in mock-transfected cells, 0.75 in poly(I:C)-transfected cells and 0.84 in SARS-CoV-2-infected cells (data not shown). Using the A549-ACE2 cells stably expressing IFI27, we observed that poly(I:C) treatment and SARS-CoV-2 infection increased the expression of MDA5 (**Figure 2B**), as expected ( (Yang et al., 2013; Liu et al., 2021) and **Figure 1F**). SARS-CoV-2 infection was confirmed by using an anti-N protein antibody (**Figure 2B**). Interestingly, we also observed a partial co-localization of IFI27 and endogenous MDA5 in poly(I:C)-transfected cells and SARS-CoV-2 infected cells (**Figure 2B**), as analyzed by immunofluorescence and confocal microscopy, supporting the interaction of IFI27 and MDA5 shown in the coimmunoprecipitation experiments (**Figure 1**).

### The protein IFI27 negatively affects MDA5 activation and the induction of innate immune responses

**Figure 3:**
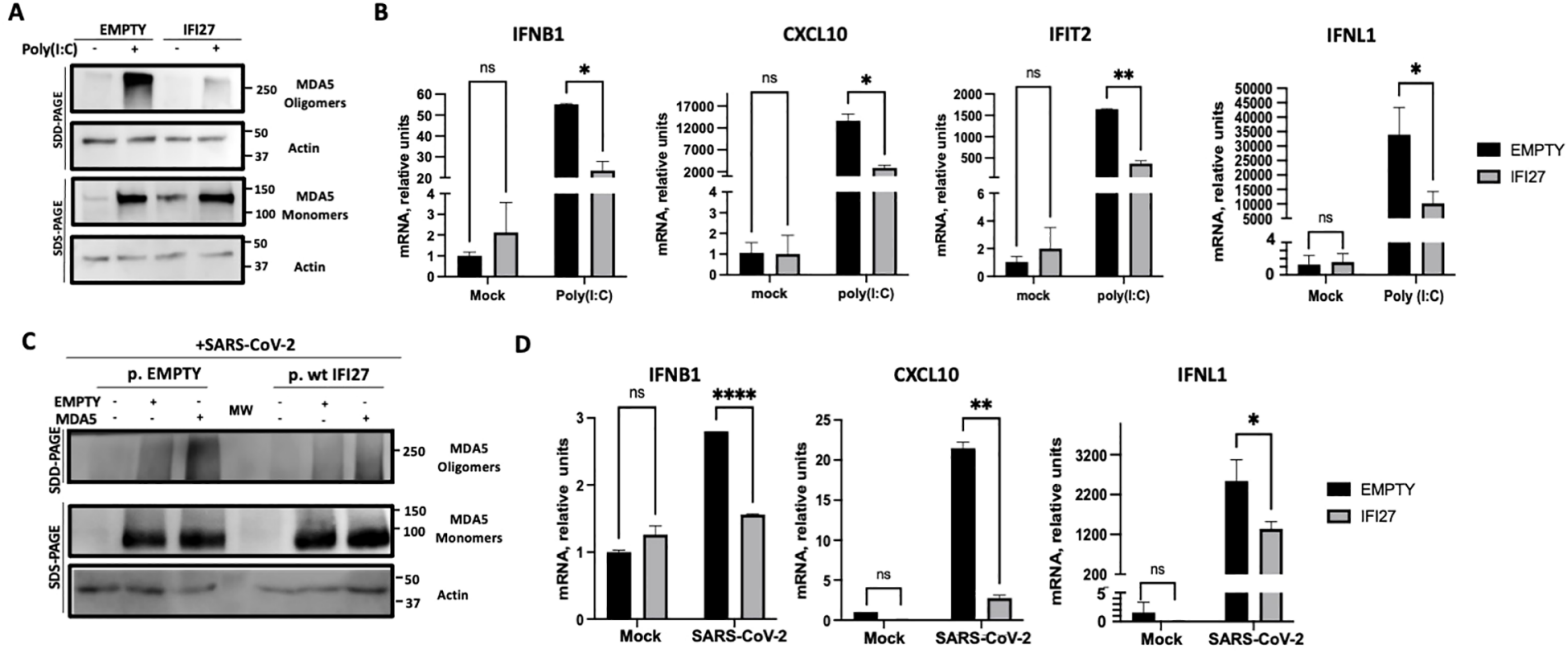
Effect of IFI27 on MDA-5 activation and the induction of innate immune responses in cells stably expressing IFI27. **(A, B)** A549-ACE2-IFI27 KO cells stably expressing IFI27 (IFI27) or control cells stably transfected with the empty plasmid (empty) were transfected with 60 ng/ml poly(I:C) for 24h or left untreated. **(A)** Western blots using anti-MDA5, and anti-actin (as loading control) specific antibodies were performed under SDD- and SDS-PAGE conditions. Molecular weight markers are indicated (in kilodaltons) on the right. **(B)** The levels of IFNB1, CXCL10, IFIT2, and IFNL1 were evaluated by RT-qPCR at 24 h after poly(I:C) transfection and compared between the A549-ACE2-IFI27 KO cells stably expressing IFI27 and the control cells transfected with the empty plasmid. Error bars represent standard deviations (SD) of results of measurements performed in triplicate wells. *p< 0.05, **p<0.01, using an Student’s t test. Ns, non-significant (p>0.05). **(C, D)** A549-ACE2-IFI27 KO cells stably expressing IFI27 or control cells stably transfected with the empty plasmid, were transfected with an MDA5-expressing plasmid or with the empty plasmid, as control, and then infected with SARS-CoV-2. **(C)** Western blots using anti-MDA5, and anti-actin (as loading control) specific antibodies were performed under SDD- and SDS-PAGE conditions. Molecular weight markers are indicated (in kilodaltons) on the right. **(D)** After SARS-CoV-2 infection, the levels of IFNB1, CXCL10 and IFNL1 in untransfected cells were evaluated by RT-qPCR and compared between the A549-ACE2-IFI27 KO cells stably expressing IFI27 and the control cells transfected with the empty plasmid. Error bars represent standard deviations (SD) of results of measurements performed in triplicate wells. *p< 0.05, **p<0.01, ***p<0.001, ****p<0.0001, using an Student’s t test. Ns, non-significant (p>0.05).

It has been shown that MDA5 detects poly(I:C) and SARS-CoV-2 infections, leading to MDA5 activation and the induction of innate immune responses (Kato et al., 2008; Dias Junior et al., 2019; Rebendenne et al., 2021; Sampaio et al., 2021; Yin et al., 2021). To first analyze whether IFI27 affects MDA5 activation, the A549-ACE2 IFI27 KO cells stably expressing IFI27-HA (A549-ACE2-IFI27 KO + IFI27-HA) and the control cells (A549-ACE2-IFI27 KO + empty), transfected with the empty plasmid, were transfected with poly(I:C) or left untransfected. A Western blot with an MDA5 antibody in native and denaturing conditions was performed. As shown in **Figure 1F**, the expression of MDA5 was induced by poly(I:C) (**Figure 3A**). Interestingly, whereas the expression of MDA5 monomers was similar in the control cells and the cells expressing IFI27-HA, the presence of MDA5 oligomers was clearly decreased in the cells overexpressing IFI27-HA, compared to the control cells (**Figure 3A**). Given that it has been shown that oligomerization of the RNA sensor MDA5 activates the antiviral innate immune responses (Jiang et al., 2012; Rehwinkel and Gack, 2020; Liu et al., 2021), these data suggest that IFI27 affects MDA5 activation. To further analyze whether IFI27 could affect MDA5 activation, the expression of the innate immune response mediators such as a type I IFN (IFNB1), whose expression is directly activated by MDA5 activation, IFIT2 (which is an ISG), a type III IFN (IFNL1), and the pro-inflammatory cytokine CXCL10 was analyzed by RT-qPCR (**Figure 3B**). As expected (Villamayor et al., 2023a, 2023b), the expression of these innate immune response genes was induced in poly(I:C)-transfected cells (**Figure 3B**). Interestingly, the expression of IFNB1, IFIT2, IFNL1 and CXCL10 was diminished in the cells overexpressing IFI27, compared to the control cells (**Figure 3B**), correlating with the decreased activation of MDA5 observed in the Western blots (**Figure 3A**). These results further corroborate our previous published results showing that IFI27 negatively modulates the innate immune responses after poly(I:C) transfection.

Given that MDA5 induces the innate immune responses after SARS-CoV-2 infection (Rebendenne et al., 2021; Sampaio et al., 2021; Yin et al., 2021), we analyzed whether IFI27 can modulate MDA5 activation after SARS-CoV-2 infection. To this end, we used the A549-ACE2-IFI27 KO + IFI27-HA and the control cells A549-ACE2-IFI27 KO +empty, and we left them mock-transfected, or transfected them with the plasmid expressing MDA5-FLAG, or an empty plasmid, as control. Then, the cells were infected with SARS-CoV-2 during 24 h. The presence of MDA5 oligomers was increased in the cells transfected with plasmids, compared to the non-transfected cells, suggesting that the plasmid transfection induces the expression of MDA5 (**Figure 3C**). Interestingly, the presence of MDA5 oligomers was decreased in the cells overexpressing IFI27 compared to the control cells (**Figure 3C**), further suggesting that IFI27 modulates MDA5 activation after SARS-CoV-2 infection. Furthermore, correlating with a higher level of MDA5 activation and higher levels of IFNB1, CXCL10 and IFNL1 in the absence of IFI27-HA overexpression in SARS-CoV-2-infected cells (**Figure 3D**), SARS-CoV-2 grew with higher titers in the cells overexpressing IFI27-HA, compared to control cells (**Supplementary Figure S2**).

**Figure 4:**
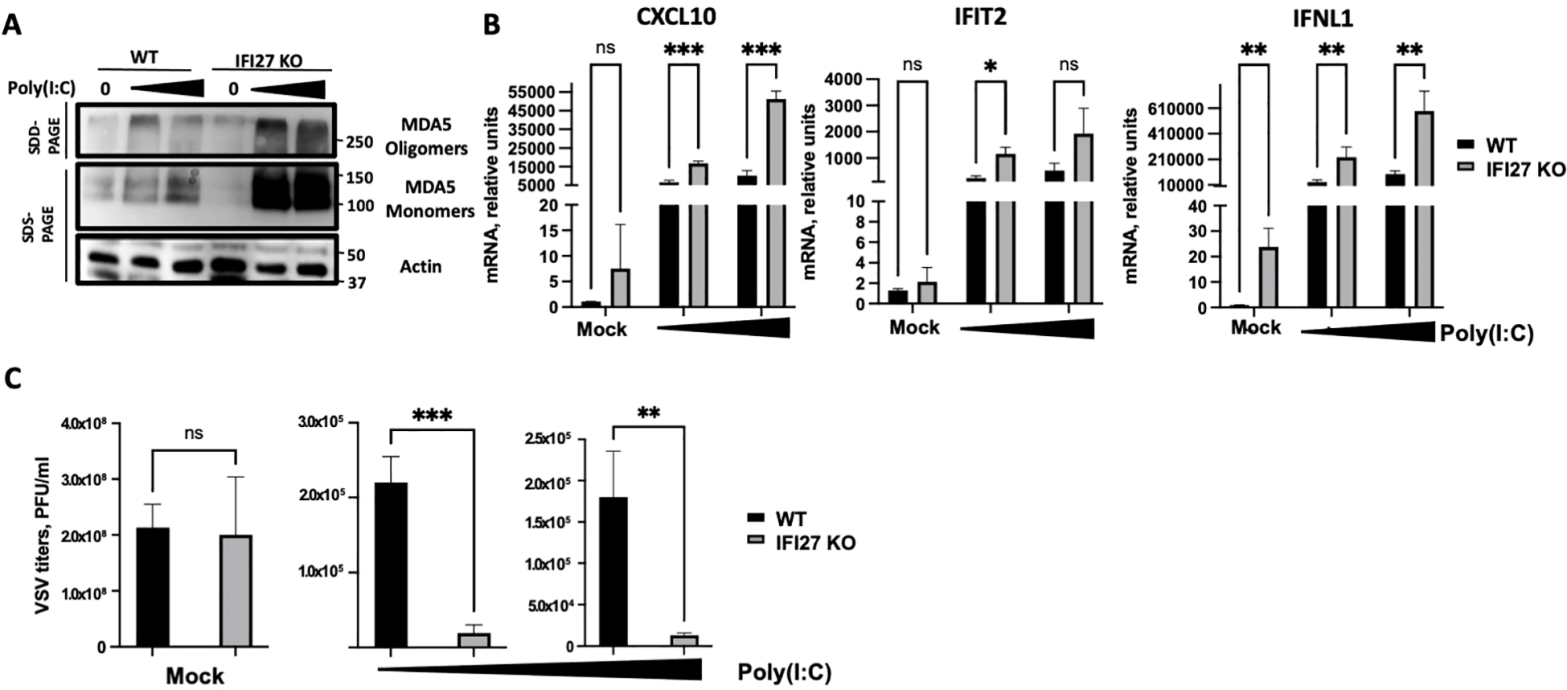
Effect of IFI27 on MDA-5 activation and the induction of innate immune responses using IFI27 KO cells. **(A–C)** A549-ACE2 WT cells and cells KO for the IFI27 gene, were transfected with two different concentrations, 5 ng/ml and 12.5 ng/ml of poly(I:C) for 24h or left untreated. **(A)** Western blots using anti-MDA5, and anti-actin (as loading control)-specific antibodies were performed under SDD- and SDS-PAGE conditions. Molecular weight markers are indicated (in kilodaltons) on the right. **(B)** The levels of CXCL10, IFIT2, and IFNL1 were evaluated by RT-qPCR after at 24 h at 5 ng/ml and 12.5 ng/ml poly(I:C) transfection and compared between the A549-ACE2 WT cells and IFI27 KO cells. Error bars represent standard deviations (SD) of results of measurements performed in triplicate wells. *p< 0.05, **p<0.01, ***p<0.001, using an Student’s t test. Ns, non-significant (p>0.05). **(C)** Mock-transfected cells or poly(I:C) -transfected cells were infected with rVSV-GFP, and viral titers were measured by plaque assays at 24 hpi. *p< 0.05, **p<0.01, ***p<0.001, using an Student’s t test. Ns, non-significant (p>0.05). Results show the mean of three independent replicates. Two independent experiments were performed with similar results.

To confirm these results using a different cellular system, A549-ACE2 parental and IFI27 KO A549-ACE2 cells, previously generated and published (Villamayor et al., 2023a) were used. Again, the cells were treated with two different concentrations of poly(I:C) and the expression of MDA5 and the presence of MDA5 oligomers were analyzed by Western blot, showing that both the expression of MDA5 and the presence of MDA5 oligomers were increased in the IFI27 KO cells, compared to the parental cells (**Figure 4A**). Correlating with the increased MDA5 activation in the IFI27 KO cells, the levels of IFIT2, IFNL1 and CXCL10 expression were increased in the IFI27 KO cells transfected with two different concentrations of poly(I:C) compared to the parental cells (**Figure 4B**), further confirming that IFI27 acts as a feedback regulator of MDA5 activation.

To investigate whether modulation of MDA5 activation by IFI27 affects subsequent antiviral IFN responses, parental A549-ACE2 cells and the IFI27 KO cells were transfected with two different concentrations of poly(I:C), as described above, to induce an antiviral state. Then, the cells were infected with a recombinant vesicular stomatitis virus (VSV) expressing the Green Fluorescent protein (GFP) (MOI 0.001) and viral titers were determined at 24 hours post-infection (hpi), as an indirect measure of the antiviral state induced in the cells, as previously shown (DeDiego et al., 2019b; Villamayor et al., 2023a, 2023b). In cells non-transfected with poly(I:C) (mock-transfected condition), high titers (>10^8^ PFU/ml) were detected, showing no significant differences in VSV-GFP titers between the WT and IFI27 KO cells (**Figure 4C**), which suggests that the function of IFI27 depends on dampening RIG-I or MDA5 signaling. Nevertheless, virus titers decreased around 100-fold in the parental cells treated with poly(I:C), compared to the non-treated cells (**Figure 4C**), negatively correlating with the induction of an antiviral state in these cells, as previously reported (DeDiego et al., 2019b; Villamayor et al., 2023a, 2023b). Interestingly, in the IFI27 KO cells transfected with poly(I:C), viral titers decreased by 11-fold, compared to the poly(I:C)-transfected A549-ACE2 parental cells (**Figure 4C**), further demonstrating that IFI27 expression negatively regulates the induction of innate immune responses.

**Figure 5:**
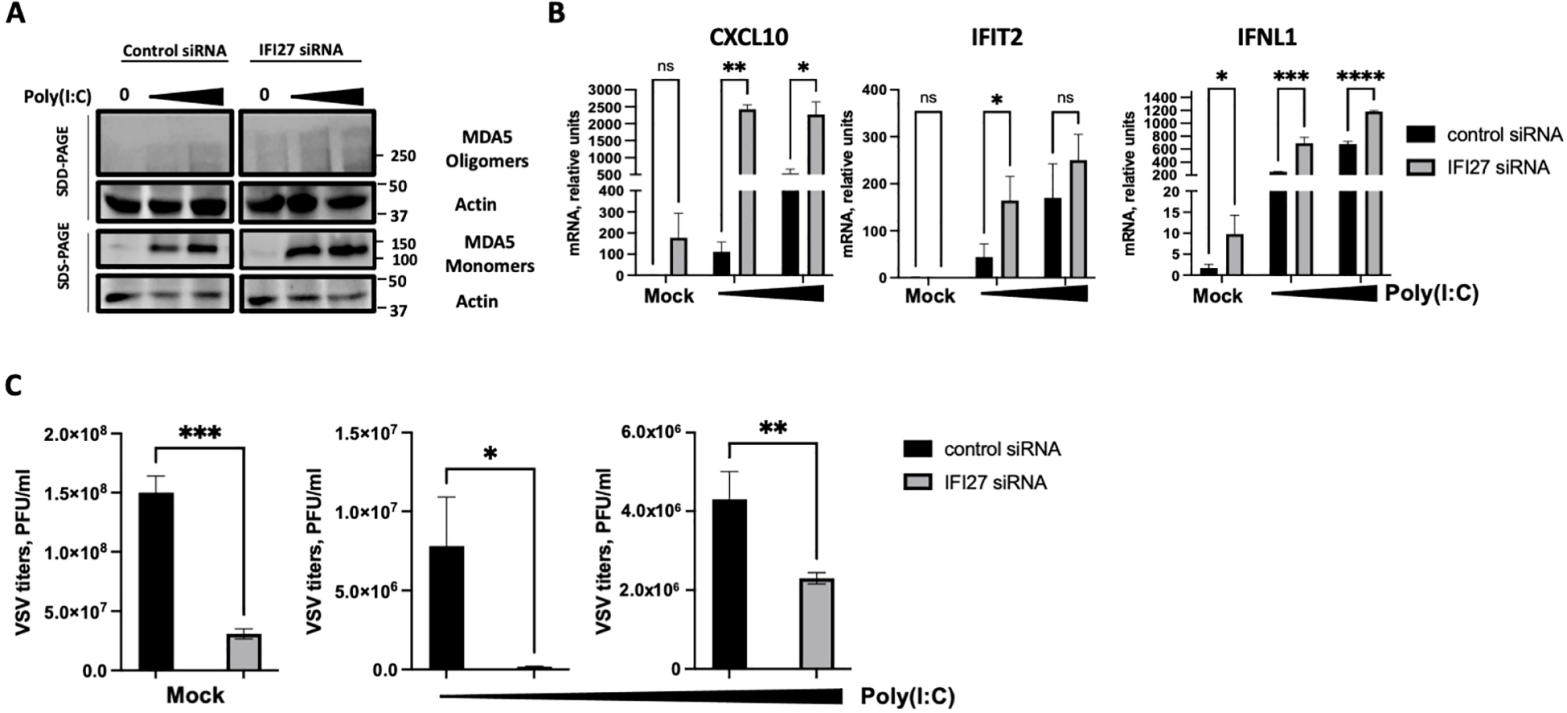
Effect of IFI27 on MDA-5 activation and the induction of innate immune responses using IFI27 KD cells. A549-ACE2 WT cells were transfected twice, 24 h apart, with a negative control siRNA, or with an siRNA specific for IFI27. At 24 h after the second siRNA transfection, the cells were treated with two different concentrations of poly (I:C) (5 ng/ml and 12.5 ng/ml respectively) for 24h or left untreated. **(A)** Western blots using anti-MDA5, and anti-actin (as loading control)-specific antibodies were performed under SDD- and SDS-PAGE conditions. Molecular weight markers are indicated (in kilodaltons) on the right. **(B)** The levels of CXCL10, IFIT2, and IFNL1 were evaluated by RT-qPCR at 24 h after poly(I:C) transfection and compared between the control cells and the cells knocked-down for IFI27. Error bars represent standard deviations (SD) of results of measurements performed in triplicate wells. *p< 0.05, **p<0.01, ***p<0.001, ****p<0.0001, using an Student’s t test. Ns, non-significant (p>0.05). **(C)** After poly(I:C) transfection, the cells were infected with rVSV-GFP, and viral titers were measured by plaque assays at 24 hpi. *p< 0.05, **p<0.01, ***p<0.001, using an Student’s t test. Results show the mean of three independent replicates. Two independent experiments were performed with similar results.

**Figure 6:**
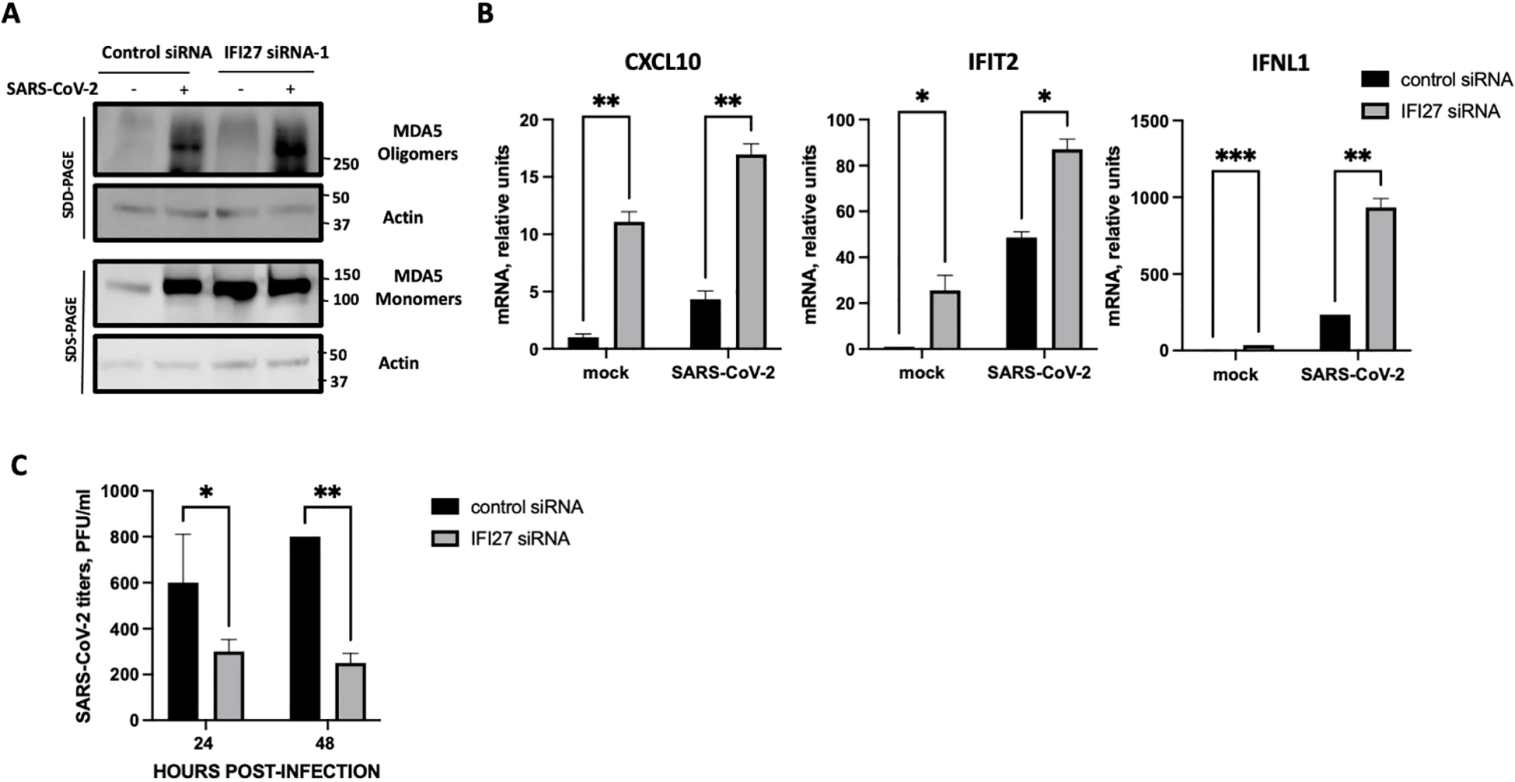
Effect of IFI27 on MDA-5 activation and the induction of innate immune responses using IFI27 KD cells. A549-ACE2 WT cells were transfected twice, 24 h apart, with a negative control siRNA, or with an siRNA specific for IFI27. At 24 h after the second siRNA transfection, the cells were infected with SARS-CoV-2 during additional 24 h. **(A)** Western blots using anti-MDA5, and anti-actin (as loading control)-specific antibodies were performed under SDD- and SDS-PAGE conditions. Molecular weight markers are indicated (in kilodaltons) on the right. **(B)** The levels of CXCL10, IFIT2, and IFNL1 were evaluated by RT-qPCR at 24 h after poly(I:C) transfection and compared between the A549-ACE2 WT cells and IFI27 KO cells. Error bars represent standard deviations (SD) of results of measurements performed in triplicate wells. *p< 0.05, **p<0.01, ***<0.001using an Student’s t test. **(C)** Viral titers at 24 hpi were evaluated by plaque assay. *p< 0.05, **p<0.01 using an Student’s t test.

As an alternative approach, IFI27 expression was knocked-down by transfecting the cells with a commercial siRNA specific for IFI27. First, we confirmed that IFI27 was efficiently silenced, by RT-qPCR, showing at least a 10-fold reduction in the IFI27 mRNA levels in the cells transfected with the siRNA specific for IFI27, compared to the control cells transfected with the non-targeted, control siRNA (**Supplementary Figure S3**). Both poly(I:C) transfection and SARS-CoV-2 infection induced the oligomerization of MDA5 (**Figures 5A**, **6A**), as expected (Lui et al., 2017). Interestingly, the presence of MDA5 oligomers was increased in the cells knocked-down for IFI27, after poly(I:C) transfection and SARS-CoV-2 infection, compared to the control cells transfected with the negative control siRNA (**Figures 5A**, **6A**), providing further evidence that IFI27 modulates MDA5 activation. Moreover, whereas the expression of IFIT2, CXCL10 and IFNL1 increased after poly(I:C) transfection and SARS-CoV-2 infection, this upregulation in the expression of these genes was further increased in the cells knocked-down for IFI27, than in the control cells (**Figures 5B**, **6B**). Furthermore, as another approach complementary to the RT-qPCRs, the cells transfected with poly(I:C) were infected with rVSV-GFP, as an indirect measure of the antiviral state induced in the cells. As we previously observed (DeDiego et al., 2019b; Villamayor et al., 2023a, 2023b), virus titers decreased around 20 and 300-fold in control cells transfected with the lower and higher poly(I:C) concentrations, respectively, compared to the non-transfected cells (**Figure 5C**), negatively correlating with the induction of an antiviral state in these cells. Interestingly, in the cells transfected with the lowest and highest poly(I:C) concentrations, knocked-down with the siRNA specific for IFI27, viral titers decreased by 46 and 2-fold, compared to the poly(I:C)-transfected control cells (**Figure 5C**), further showing that IFI27 inhibits the innate immune responses.

### IFI27 competes with MDA5 for the binding to poly(I:C)

**Figure 7:**
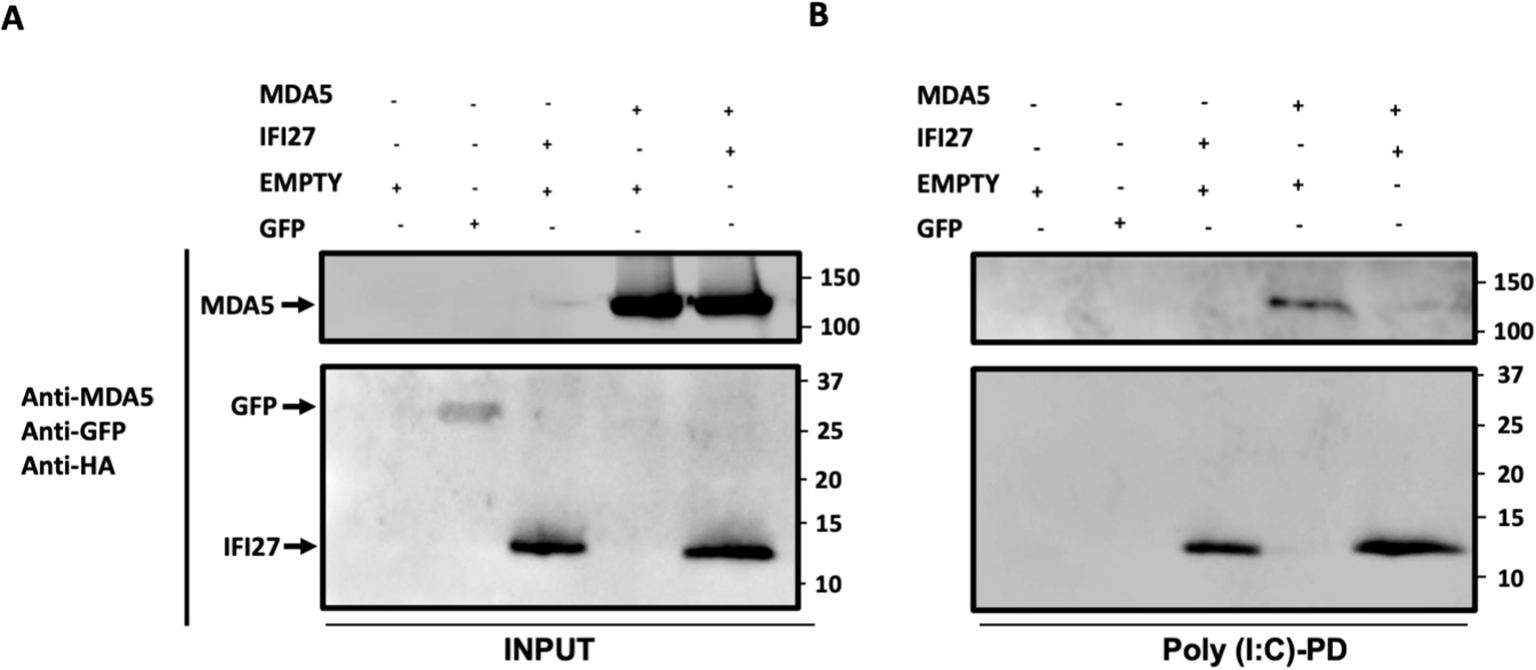
IFI27 competes with MDA5 for poly(I:C) binding. **(A, B)** Human 293T cells were transiently transfected with the pCAGGS plasmids encoding GFP, IFI27-HA, MDA5-FLAG, MDA5-FLAG plus IFI27-HA, or with an empty plasmid. Pull-down (PD) experiments using poly(I:C)-conjugated agarose beads were performed using cellular extracts. Western blotting using Abs specific for MDA5, GFP, and the HA tag (to detect IFI27), in the same blot, was performed to detect protein in the cellular lysates (Input) **(A)** and after the pull-down (poly(I:C)-PD) **(B)**. Molecular weight markers are indicated (in kilodaltons) on the right.

As both MDA5 (Kato et al., 2008; Dias Junior et al., 2019), and IFI27 (Villamayor et al., 2023a) bind to poly(I:C), and our data indicate that IFI27 negatively modulates MDA5 activation, we hypothesize that IFI27 could be competing with MDA5 for poly(I:C) binding, therefore decreasing MDA5 activation. To this end, cellular extracts overexpressing MDA5, IFI27, MDA5 plus IFI27, and GFP, as a negative control (**Figure 7A**), were incubated with agarose beads conjugated to poly(I:C), and the proteins binding to poly(I:C) were detected by Western blot. As expected, GFP was not pulled-down using poly(I:C)-conjugated agarose beads (**Figure 7B**). In contrast, MDA5 bound to poly(I:C)-agarose beads (**Figure 7B**), confirming previous reports indicating that MDA5 binds to poly(I:C) (Kato et al., 2008; Dias Junior et al., 2019). Whereas the amount of MDA5 was similar when MDA5 was expressed alone and when MDA5 was expressed in combination with IFI27 (**Figure 7A**), remarkably, the amount of MDA5 bound to poly(I:C) was lower when IFI27 was expressed, than when IFI27 was not expressed (**Figure 7B**). These data strongly suggest that IFI27 competes with MDA5 for poly(I:C) binding, providing a likely explanation for the effect of IFI27 in inhibiting MDA5 activation.

## Discussion

In this work we find that IFI27 binds to MDA5, and negatively modulates MDA5 activation, more likely by competing with MDA5 for viral RNA binding. MDA5 is a key protein sensing SARS-CoV-2 and other RNA virus infections, leading to the induction of innate immune responses. After viral RNA recognition, MDA5 changes its conformation, oligomerizes and interacts with MAVS, also known as cardif, IPS-1 and VISA, favoring the activation of the transcription factors IRF-3, IRF-7 and NF-κB, which lead to the expression of multiple proinflammatory cytokines, IFNs and ISGs (Rehwinkel and Gack, 2020). Therefore, as IFI27 is a protein localized to the mitochondria (Cheriyath et al., 2011; Jin et al., 2018), where the interaction of oligomerized MDA5 with MAVS occur (Rehwinkel and Gack, 2020), these data favor an interaction of IFI27 with MDA5 (**Figures 1**, **2**).

Although innate immune responses inhibit viral infections, exacerbated innate immune responses after viral infections could be detrimental to the host and feedback mechanisms are needed to go back to the steady state once the infections are cleared (Komuro et al., 2008; Richards and Macdonald, 2011). Therefore, in this sense, IFI27 could aid in these processes, acting to evade the host innate immune response.

IFI27 expression is induced after the infections by many viruses, such as SARS-CoV-2 (Mick et al., 2020; Gupta et al., 2021; Huang et al., 2021; Kulasinghe et al., 2022; Shojaei et al., 2022; Villamayor et al., 2023a), as part of the innate immune response. Nevertheless, as an excessive antiviral signaling can be detrimental to the host, many host factors play a role in negatively modulating innate immune responses. In this sense, we previously described that the ISGs IFI6, IFI27, IFI44 and IFI44L display feedback regulatory functions (DeDiego et al., 2019b, 2019a; Villamayor et al., 2023a, 2023b), and others have described that other ISGs, such as IFI35 and ISG56/IFIT1 negatively regulate antiviral responses as well (Li et al., 2009; Das et al., 2014). Correlating with the effect of IFI27 in decreasing innate immune responses, we have shown that IFI27 binds the PRR MDA5 in poly(I:C)-transfected and SARS-CoV-2-infected cells (**Figure 1**), decreasing its activation (**Figures 3**, **4**, **5**, **6**). Furthermore, we have shown that IFI27 competes with MDA5 for poly(I:C) binding (**Figure 7**), providing an explanation for the effect of IFI27 on impairing MDA5 activation. The data indicating that IFI27 negatively affects MDA5 activation after SARS-CoV-2 infection and poly(I:C) transfection, suggests that this effect is likely broader, impairing MDA5 activation after different viral infections. Similarly, other host proteins affect MDA5 activation. For example, the helicase DHX29 functions as an RNA co-sensor for MDA5-mediated antiviral immunity (Zhu et al., 2018). Interestingly, recent studies showed that PACT promotes MDA5 activation by facilitating its ability to oligomerize (Lui et al., 2017). The zinc-finger protein ZCCHC3 has recently been shown to function as a co-receptor for both RIG-I and MDA5, since ZCCHC3 binds with its C-terminal zinc finger domains to RNAs that activate RLRs, whereas its N-terminal domain interacts with the helicase and CTD of RIG-I and MDA5 (Lian et al., 2018).

Furthermore, MDA5 activity is modulated by post-translational modifications. Aberrant activation of MDA5 in uninfected cells is prevented by phosphorylation at S88 and S828. Upon virus infection, MDA5 is activated by removal of these phosphorylation events by PP1α/γ (Wies et al., 2013). It was recently shown that the helicase domain of MDA5 (specifically K743) is modified with K63-linked ubiquitin chains synthesized by TRIM65, which activates MDA5 by promoting its oligomerization (Lang et al., 2017). SUMOylation of RIG-I and MDA5 by TRIM38 in uninfected cells, or at early times after RNA virus infections, stabilizes the RLRs, preventing their K48-polyubiquitin-dependent degradation. However, during the late phase of infection, de-SUMOylation of RIG-I and MDA5 by sentrin/SUMO-specific protease 2 (SENP2) triggers their proteasomal degradation, aiding in finishing the pro-inflammatory response once the virus has been cleared (Hu et al., 2017). Furthermore, it has been shown that the MDA5 CARD domains get ISGylated, contributing to MDA5 activation (Liu et al., 2021).

Given the MDA5 role in recognizing viral infections and inducing antiviral responses, many viruses encode viral proteins impairing MDA5 activation. In the case of SARS-CoV-2, at least two viral proteins counteract MDA5 activation. The ISG15-dependent activation of MDA5 is antagonized through direct MDA5 de-ISGylation mediated by the papain-like protease (PLpro) (Liu et al., 2021). In addition, SARS-CoV-2 Nsp8 suppresses MDA5 antiviral immune responses by impairing the MDA5 K63-linked polyubiquitination mediated by TRIM4 (Zhang et al., 2023). Therefore, the effect of IFI27 on diminishing MDA5 activation may be even more prominent in cells infected with other viruses not encoding viral proteins counteracting MDA5 activation.

Previously we showed that IFI27 positively affects SARS-CoV-2 and IAV replication by negatively modulating the antiviral responses induced after viral infections (Villamayor et al., 2023a). Furthermore, we observe decreased VSV titers in poly(I:C)-transfected cells knocked-out for IFI27 compared to parental poly(I:C)-transfected control cells (**Figure 4C**) and in cells knocked-down for IFI27 compared to the control cells (**Figure 5C**). The effect of IFI27 in facilitating viral replication is likely mediated by negative modulation of innate immune responses as it was previously shown that VSV infection is affected by the previous antiviral states induced in the cells. Similarly, silencing other ISGs such as IFI35 and ISG56/IFIT1 proteins, which negatively modulate IFN responses, decreases VSV replication (Li et al., 2009; Das et al., 2014).

## Data Availability

The raw data supporting the conclusions of this article will be made available by the authors, without undue reservation.

## Supplementary material

Supplementary Figure 1 Generation of cells stably expressing IFI27. A549 cells were transfected with the pCAGGS plasmid expressing IFI27 fused to an HA tag (pCAGGs-IFI27-HA) or with the empty plasmid as control, and the transfected cells were selected with the antibiotic hygromycin. The cells were fixed with paraformaldehyde, and IFI27-HA was labeled with an anti-HA (to detect IFI27) specific antibody (in green), and nuclei were stained with DAPI (in blue). Scale bar, 10 μm.

Supplementary Figure 2 Effect of IFI27 overexpression on SARS-CoV-2 replication. A549-ACE2-IFI27 KO cells stably expressing IFI27 or control cells, and control cells, stably transfected with the empty plasmid were infected with SARS-CoV-2. Viral titers were measured by a lysis plaque assay at 24 hpi. Results show the mean of three independent replicates. Two independent experiments were performed with similar results.

Supplementary Figure 3 Efficiency of IFI27 siRNAs. Human A549 cells were transfected with a control, non-targeted siRNA or with an siRNA specific for IFI27. Then, the cells were left mock-transfected or transfected with two different concentrations of poly(I:C) as in **Figure 5**. At 24 after poly(I:C) transfection, total RNAs were purified and used to determine the mRNA levels for IFI27 by RT-qPCR. *p< 0.05, **p<0.01, using an Student’s t test.
