# Supplementary figures and images for: The IFN-induced protein IFI27 binds MDA5 and counteracts its activation after SARS-CoV-2 infection
Source: Front Cell Infect Microbiol. 2024 Oct 4;14:1470924. doi: 10.3389/fcimb.2024.1470924 (PMC11486742; doi:10.3389/fcimb.2024.1470924)

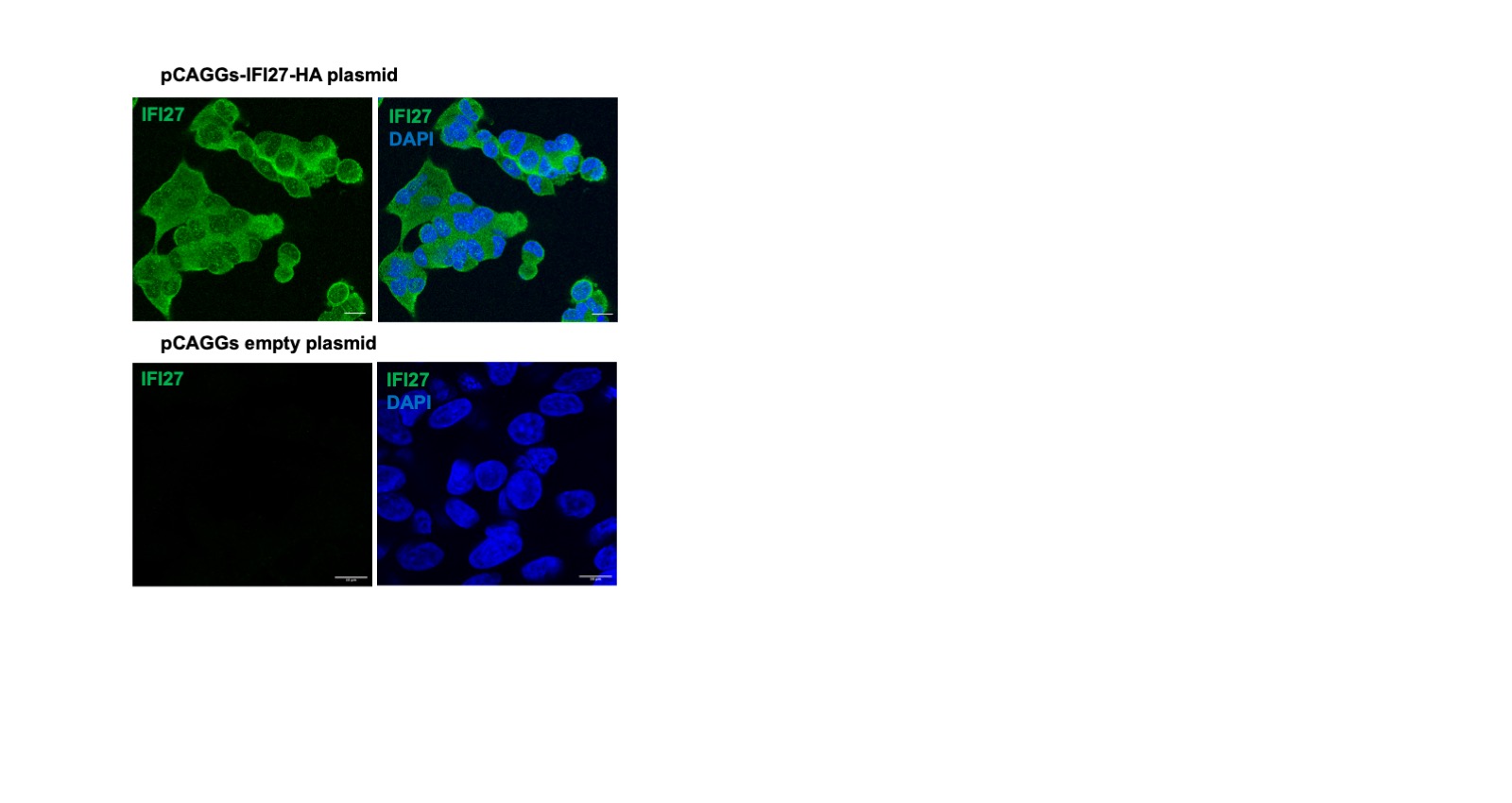

Supplement: Supplementary Figure 1 — Generation of cells stably expressing IFI27. A549 cells were transfected with the pCAGGS plasmid expressing IFI27 fused to an HA tag (pCAGGs-IFI27-HA) or with the empty plasmid as control, and the transfected cells were selected with the antibiotic hygromycin. The cells were fixed with paraformaldehyde, and IFI27-HA was labeled with an anti-HA (to detect IFI27) specific antibody (in green), and nuclei were stained with DAPI (in blue). Scale bar, 10 μm. [file Image1.jpeg]

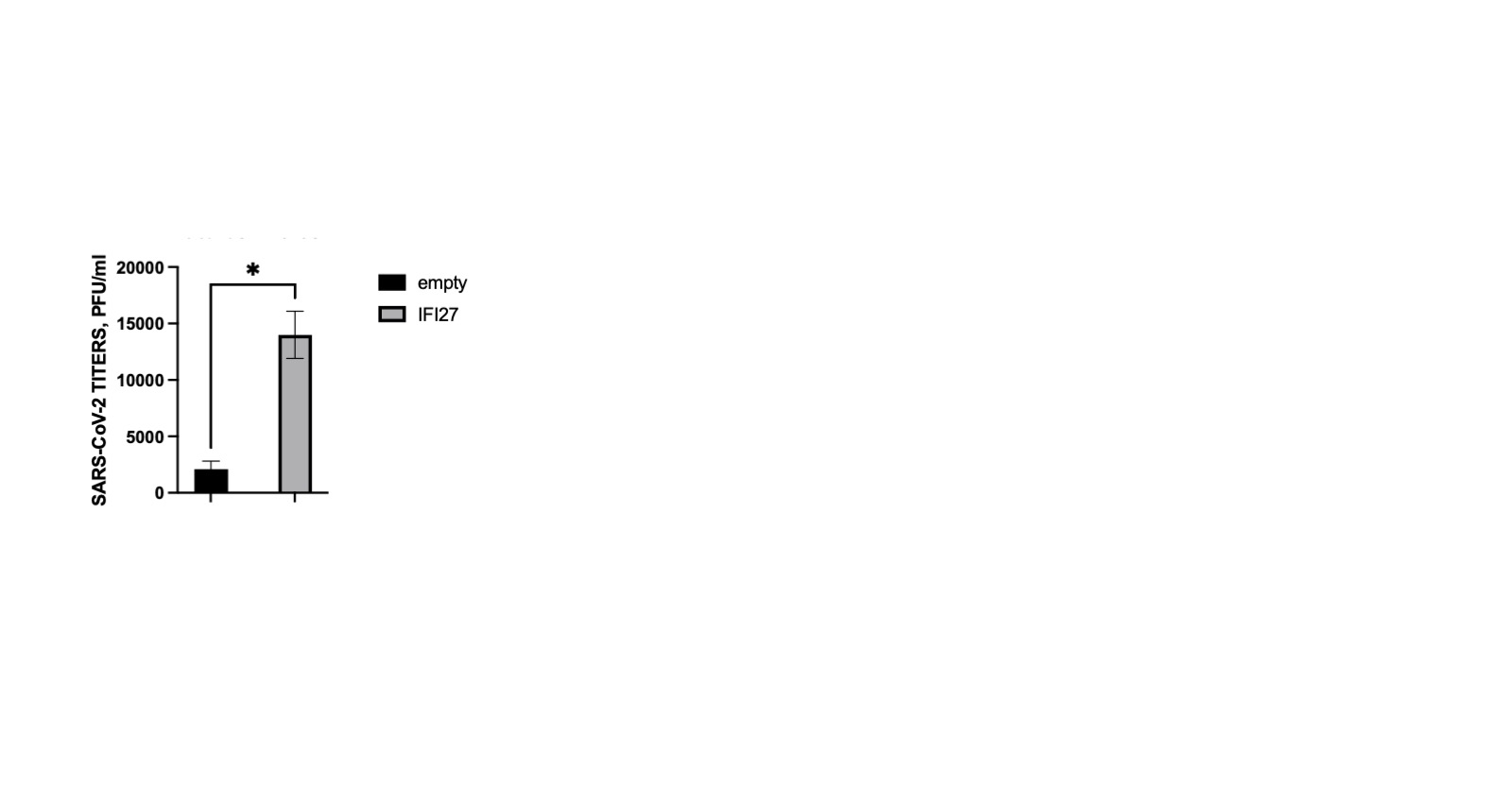

Supplement: Supplementary Figure 2 — Effect of IFI27 overexpression on SARS-CoV-2 replication. A549-ACE2-IFI27 KO cells stably expressing IFI27 or control cells, and control cells, stably transfected with the empty plasmid were infected with SARS-CoV-2. Viral titers were measured by a lysis plaque assay at 24 hpi. Results show the mean of three independent replicates. Two independent experiments were performed with similar results. [file Image2.jpeg]

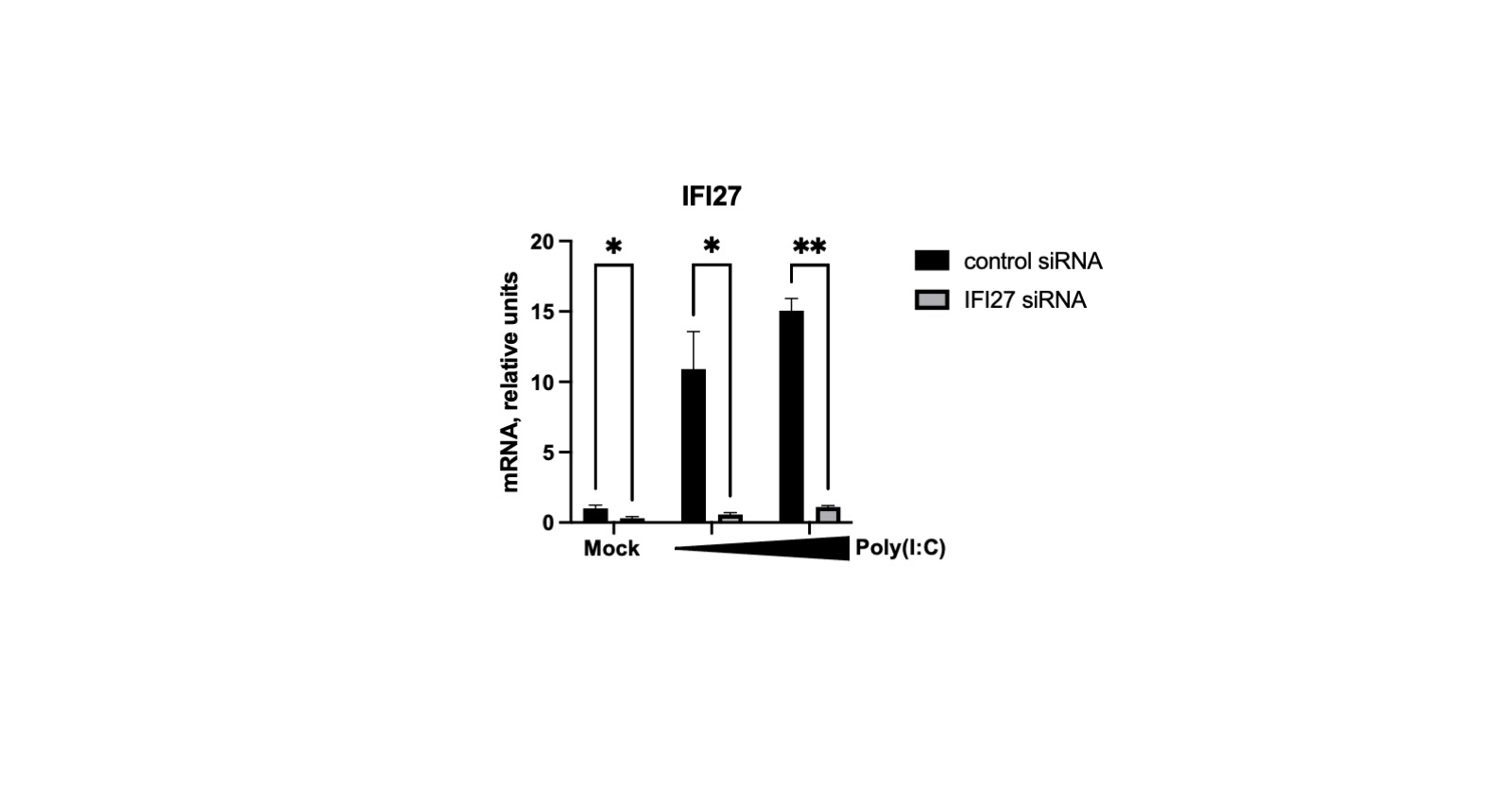

Supplement: Supplementary Figure 3 — Efficiency of IFI27 siRNAs. Human A549 cells were transfected with a control, non-targeted siRNA or with an siRNA specific for IFI27. Then, the cells were left mock-transfected or transfected with two different concentrations of poly(I:C) as in Figure 5 . At 24 after poly(I:C) transfection, total RNAs were purified and used to determine the mRNA levels for IFI27 by RT-qPCR. *p< 0.05, **p<0.01, using an Student’s t test. [file Image3.jpeg]
